# p53 in the mitochondria, as a trans-acting protein, provides error-correction activities during the incorporation of non-canonical dUTP into DNA

**DOI:** 10.18632/oncotarget.12331

**Published:** 2016-09-28

**Authors:** Elad Bonda, Galia Rahav, Angelina Kaya, Mary Bakhanashvili

**Affiliations:** ^1^ Infectious Diseases Unit, Sheba Medical Center, Tel Hashomer 5265601, Israel; ^2^ The Mina and Everard Goodman Faculty of Life Sciences, Bar-Ilan University, Ramat-Gan, Israel

**Keywords:** p53, mitochondria, uracil, DNA synthesis, exonuclease

## Abstract

Mutations in mitochondrial DNA is an outcome of errors produced by DNA polymerase γ during replication and failure of the repair mechanism. Misincorporation of non-canonical dUTP leads to mutagenesis or apoptosis, and may contribute to the cytotoxic effects of 5′-fluorouracil chemotherapy. Tumor suppressor p53 protein in the mitochondria displays physical and functional interactions with mitochondrial DNA and polymerase γ, and by its intrinsic 3′→5′ exonuclease activity can diminish the polymerization errors. Here we demonstrate the impact of p53 on incorporation of uracil into DNA examined with mitochondrial fractions, as the source of polymerase γ. p53 in mitochondria facilitates DNA damage repair functions resulting from uracil–DNA misincorporation. Our biochemical studies revealed that the procession of U:A and mismatched U:G lesions enhances in the presence of recombinant or endogenous cytoplasmic p53. p53 in mitochondria can function as an exonuclease/proofreader for polymerase γ by either decreasing the incorporation of non-canonical dUTP into DNA or by promoting the excision of incorporated nucleotide from nascent DNA, thus expanding the spectrum of DNA damage sites exploited for proofreading as a trans-acting protein. The data suggest that p53 may contribute to defense of the cells from consequences of dUTP misincorporation in both normal and tumor cells.

## INTRODUCTION

A high frequency of mutations within mitochondrial DNA (mtDNA), resulting in mitochondrial dysfunctions, are an important source of various diseases including cancer and human aging [1, 2]. MtDNA damage yield inaccurate translation and synthesis of respiratory chain enzymes imperative for energy production in the mitochondrial inner membrane [3]. A “mutator” phenotype of the mitochondrial genome is a phenomenon caused by incorporation of incorrect nucleotide, errors produced due to the presence of unbalanced dNTP concentrations and oxidative mutagenesis [2, 3]. Mitochondrial DNA polymerase γ (pol γ), a singular replicase responsible for the biogenesis of mtDNA, consists DNA polymerase and exonuclease activities [4]. The mutagenic mechanisms are related to replication errors formed by pol γ during DNA synthesis by misinsertion of wrong nucleotide or by diminished proofreading capacity [4, 5]. Remarkably, mice expressing an exonuclease-deficient pol γ exert a 3–5-fold increase in the level of point mutations and undergo enhanced aging [6]. MtDNA is not protected by histones and mtDNA repair is ineffective [1]. To certify mtDNA integrity, cells contain quality control mechanisms and DNA damage response pathway(s) comprising mtDNA replication/repair preservation programs that either preclude or repair damage [7]. Mitochondrial context is affected by cellular stresses. MtDNA integrity and replication rest on numerous nucleus-encoded DNA repair proteins normally localized in mitochondrial matrix or imported upon various stress signals [8]. Tumor suppressor p53 protein is involved in diverse cellular processes including cell cycle arrest (thus preventing the replication of damaged DNA), apoptosis (for eliminating defective cells), or DNA-damage repair [9–10]. p53 protein executes multi-compartmental functions in the cell by either a numerous p53-regulated proteins and/or by its intrinsic biochemical activities [11, 12]. p53, by providing exonuclease activity, enables error-correction thus functioning as a guardian of genome [12, 13]. In addition to regulation of apoptosis by p53 via protein-protein interaction at the mitochondrial outer membrane, the biological routes regulated by p53 comprises mtDNA synthesis/repair for mitochondrial function after translocating to matrix [14]. RECQL4 was detected essential for the transport of p53 to mitochondria [15, 16]. The multi-functional p53 protein promotes DNA repair both directly or indirectly through multiple mechanisms [13]. Mitochondrial p53 levels are proportional to total p53 levels and the majority of p53 was present inside the intra-mitochondrial compartment-matrix [17]. Co-localization of p53 with mtDNA, pol γ and various constituents of the mtDNA replication apparatus (*e.g.* transcription factor A-TFAM and single-stranded DNA-binding proteins), even in the absence of exogenous stress independent of apoptosis, establishes a non-apoptotic function for matrix-localized p53 which underlines an importance of p53 in mtDNA homeostasis [18–20]. Several studies illustrated the participation of p53 in mtDNA repair in a variety of systems: a)p53 enhances base excision repair through direct interaction with the repair complex in mouse liver and cancer cells [21]. b) Intra-mitochondrial p53 provides an error-repair proofreading function for pol γ by excision of misincorporated nucleotides [22]. c)p53 is proficient of hydrolyzing the 8-oxo-7,8-dihydro-2′-deoxy-guanosine (8-oxodG) present at the 3′-end of DNA, a well-known marker of oxidative stress [20]. d)p53 regulates mtDNA copy number, which may impact mitochondrial and cellular functions [23]. Apparently, the functional interaction of p53 and pol γ is significant for avoiding mtDNA mutations and mtDNA depletions that are frequently observed in human cancers and neurodegenerative diseases [13].

Uracil (dU) in DNA, resulting from spontaneous cytosine deamination and/or incorporation of non-canonical dUTP during replication, leads to mutagenesis and apoptosis [24, 25]. The frequency of dU incorporation depends upon the relative intracellular pool size of dUTP and dTTP [26]. The enzyme deoxyuridine triphosphate nucleotidohydrolase (dUTPase), which facilitates the conversion of dUTP to dUMP further utilized by thymidylate synthase (TS) for synthesis of dTMP, may avoid misincorporation of dU into DNA by decreasing the dUTP/dTTP ratio [27]. The presence of dUTPase in nuclei and mitochondria points that keeping a low level of uracil in DNA is important for the integrity of nuclear as well as mitochondrial DNA [27]. Remarkably, the expression of the nuclear isoform of dUTPase is predominantly cell-cycle and proliferation-dependent, whereas the mitochondrial isoform is constitutively expressed [27].

The misincorporation of dU, as a result of accumulation of dUTP, plays a critical role in cytotoxicity mediated by TS inhibitors, such as the commonly used anticancer drug 5-fluorouracil (5-FU) [28]. DNA directed cytotoxicity of chemotherapeutic agents (*e.g*.5-FU) not only depends on accumulation of dUTP, but may also be determined by the efficiency of the DNA repair mechanisms (*e.g*. excision repair, mismatch repair) which preclude the incidence of the mistake [29]. Pol γ is incapable to readily correct U:A mismatches [26]. Of note, an augmented levels of mitochondrial p53 was reported in response to 5-FU [30]. Given the functional cooperation between pol γ and p53 in mitochondria, we aimed to ascertain the impact of p53 during the incorporation of dU into DNA. Our biochemical studies revealed that p53 in mitochondria can function as an exonuclease/proofreader for pol γ by either decreasing the incorporation of non-canonical dUTP into DNA or by promoting the excision of incorporated dU from DNA, thus substantiating and expanding the role of p53 in DNA damage repair.

## RESULTS

### Incorporation of dUTP by pol γ in mitochondria

**Figure 1:**
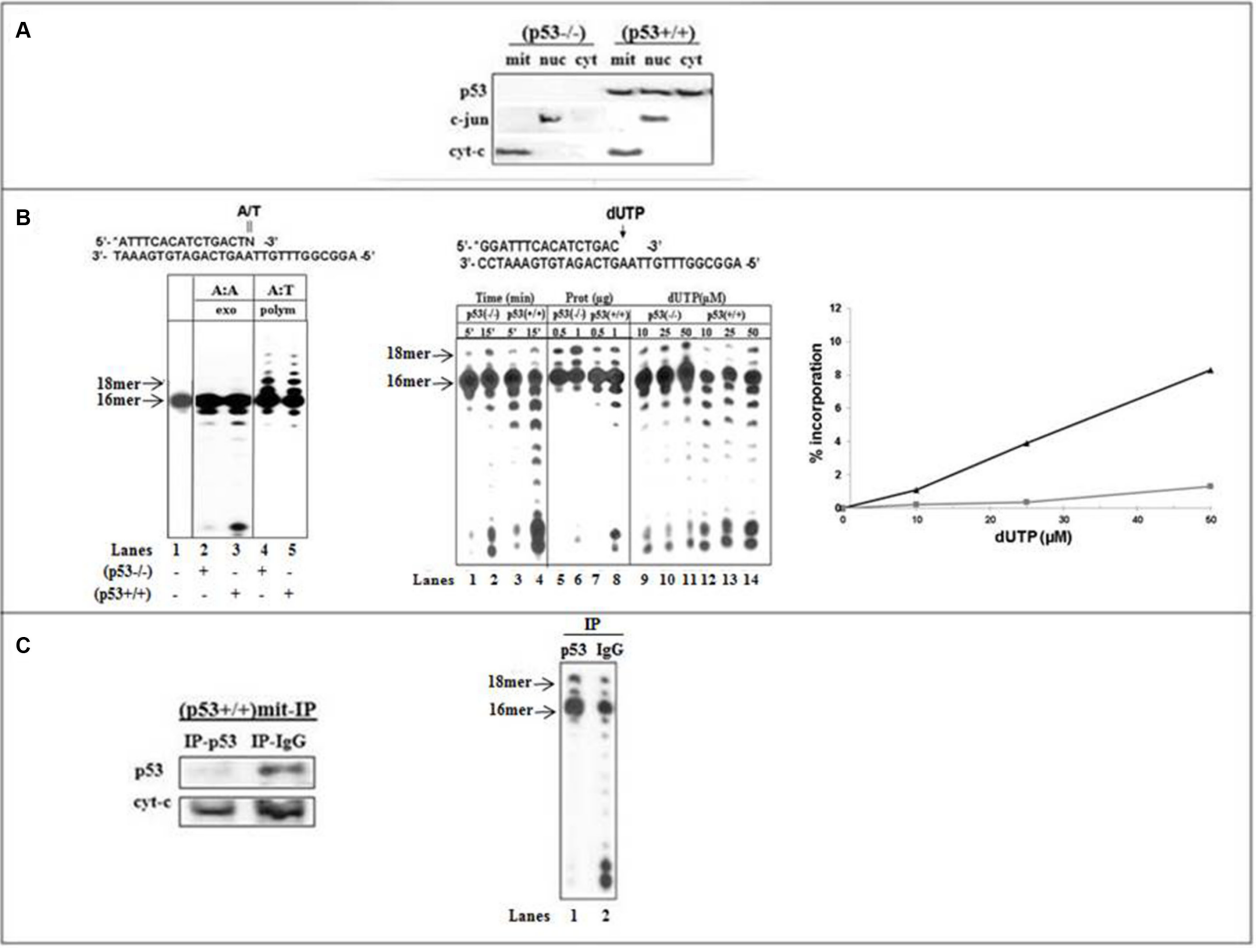
Incorporation and excision of wrong nucleotide in mitochondrial fractions of HCT116 cells (**A**) Analysis of p53 levels in subcellular compartments of HCT116 cells by Western blotting. Protein samples (10 μg) from mitochondrial (mit), nuclear (nuc) and cytoplasmic (cyt) fractions were subjected to SDS-PAGE. p53 protein expression was detected by the Do-1 anti human p53 mAb. The distribution of the nuclear marker c-jun or the mitochondrial marker cyt-C was analyzed to ascertain the purity of each fraction. (**B**) Left: The exonuclease activity (lanes 2 and 3) or the DNA polymerization (lanes 4 and 5) was examined with dsDNA substrate (lane 1) containing 3′-terminal A:A mispair (N = A) or correct A:T base pair (N = T) with mitochondrial fractions of (p53−/−) (lanes 2 and 4) or (p53+\+) (lane 3 and 5) cells. After 10-min incubation at 37°C, 5 μl aliquots were withdrawn and analyzed on 16% polyacrylamide gel, as described in Materials and Methods. The positions of the 16- and 18 mer products are indicated by arrows. Middle-The incorporation of dUTP opposite the template A was examined with mit(p53−/−) or mit(p53+\+) by increasing the incubation time (lanes 1–4), the amount of mitochondrial protein extracts (lanes 5–8), or the concentration of dUTP (lanes 9–14). Right-Incorporation rates were determined for mit(p53−/−) (■) and mit(p53+\+) (▲) detected in the presence of various concentrations of dUTP from Figure 1B-middle (lanes 9–14), by measuring the radioactivity associated with the extension products by densitometric analysis after scanning the autoradiograms and were expressed as the percentage of the total input radioactivity. (**C**) Left-The expression of p53 was tested in mitochondrial fractions of HCT116(p53+/+) cells depleted by either Do-1 anti-p53 antibodies (IP-p53) or non-specific anti-IgG antibodies (IP-IgG). Right-The incorporation of dUTP opposite the template A was assessed with (p53+\+)mit depleted by Do-1 anti-p53 Abs (lane 1) or anti-IgG Abs (lane 2). Aliquots taken were analyzed by PAGE. The positions of the 16- and 18 mer products are indicated by arrows.

The functional interaction between p53 and pol γ during incorporation of dU was assessed during DNA synthesis catalyzed by the replication machinery of mitochondrial (mit) fractions derived from isogenic HCT116 cells, as a model system [22]. Western blot analysis validated the location of endogenous p53 in mit(p53+/+) but not in mit(p53−/−) (Figure 1A). An antibody directed against cytochrome c was used to control for the enrichment of the mitochondrial fraction and to serve as a reference when comparing p53 levels between extracts. The absence of c-jun nuclear marker protein confirmed the purity of lysates, thus excluding the possibility that the polymerization or excision activity detected in mitochondrial fractions, exploited as a the source of pol γ, might be due to a contamination of the extracts with nuclear DNA polymerases and exonucleases.

We designed dsDNA substrates containing 3′-terminal correct A_t_:T pair or A_t_:A mispair. The sequences of the substrates were optimized to provide an AT-rich surrounding region (A:T versus G:C rich sequences) of the target site to maximize efficient proofreading in adjacent to relatively unstable DNA regions [31]. This point assists to accentuate the capability of p53 to accomplish effective error correction. The mitochondrial lysates of (p53+/+) and (p53−/−) cells expressing equivalent DNA polymerization activity (implying similar levels of pol γ) tested for exonuclease activity showed that under non-polymerization conditions the 3′-terminal A:A mispair excision with mit(p53+/+) (Figure 1B-left, lane 3) was more efficient than with mit(p53−/−) (lane 2). Since both fractions had comparable DNA polymerization activities (lanes 4 and 5), it is apparent that the observed efficient exonuclease activity in mit(p53+/+) was not due to a high level of pol γ. Apparently, the presence of protein with an intrinsic exonuclease activity in p53-harboring extracts may account for the efficient excision activity.

Next, the incorporation of dU opposite template A was examined with standing-start dsDNA substrate (wherein the target template residue immediately follows the 3′-terminal end of the primer) [31] (Figure 1B-middle). The production of 17- and 18 mer products following the generation of the A_t_:U pairs with mit(p53−/−) was related to the increase the time of incubation (lanes 1–2), the amount of extracts (lanes 5–6), or the concentration of dUTP (lanes 9–11). The results exhibited that incorporation of dU with mit(p53+/+) (lanes 3–4, 7–8, 12–14) was lower compared to that with mit(p53−/−) (lanes 1–2, 5–6, 9–11), as reflected by the decrease in the intensity of 17- and 18 mer products. Four separate preparations of mitochondrial extracts each tested for the markers establishing no contamination of nuclear proteins, yielding the similar results. Quantitative analysis further validates the lower (about 8.5-fold) insertion of dU into DNA with mit(p53+/+) related to that with mit(p53−/−) (Figure 1B-right).

To ensure the contribution of endogenous p53 to error correction in mit(p53+/+) we explored immuneprecipitation experiments from repair-proficient lysates (Figure 1C-left). The inhibitory effect perceived with mit(p53+/+) was significantly attenuated by the specific depletion of p53 by Do-1 anti-p53 antibodies (Figure 1C-right, lane 1) while being un-affected by non-specific depletion by the anti-IgG antibodies, that did not affect neither p53 expression nor the extent of decrease in A_t_:U pair production noticed with mit(p53+/+) (lane 2). Notably, specific depletion of p53 did not affect polymerization activity (data not shown). Based on observed correlation between the existence of p53 in mitochondria, augmented excision activity and low efficiency of A_t_:U pair generation, p53 may be a plausible candidate for processing of this type of DNA lesion.

**Figure 2:**
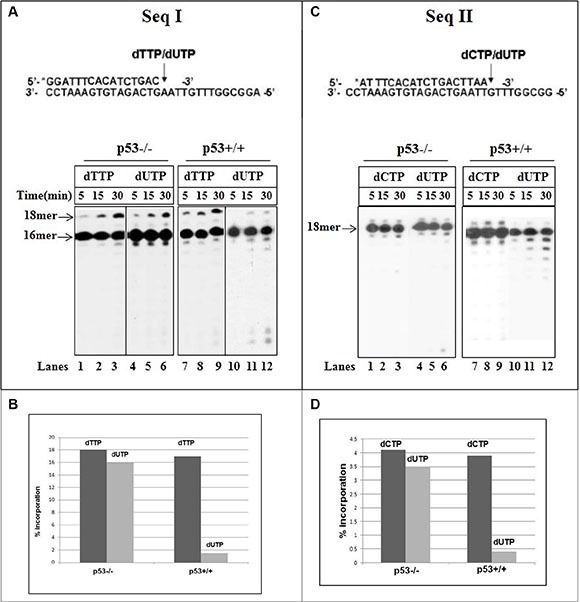
Incorporation of dUTP into DNA with mitochondrial fractions of HCT116 cells (**A**) Time course of incorporation of correct dTTP (0.05 mM) or wrong dUTP (0.05 mM) opposite template A (sequence I) was tested with mit(p53−\−) (lanes 1–6) or mit(p53+\+) (lanes 7–12). (**B**) Primer extension rates were determined from Figure 2A by measuring the radioactivity associated with the extension products, expressed as the percentage of the total input radioactivity, detected during incorporation of dTTP or dUTP. (**C**) Time course of incorporation of correct dCTP (0.05 mM) or wrong dUTP (1 mM) opposite template G (sequence II) was analyzed with mit(p53−\−) (lanes 1–6) or mit(p53+\+) (lanes 7–12). Aliquots were taken at various times and analyzed by PAGE. (**D**) Primer extension rates were determined from Figure 2C by measuring the radioactivity associated with the extension products, expressed as the percentage of the total input radioactivity, detected during incorporation of dCTP or dUTP.

DNA pol γ can utilize either dTTP or dUTP as a precursor for DNA synthesis [32]. We have assessed DNA synthesis with mit(p53−/−) in reactions where constant level (50μM) of dTTP or dUTP was supplemented individually into separate reactions. There was no significant variance in the efficiency of incorporation between dTTP and dUTP (Figure 2A, Seq I) (lanes 1–6). In contrast, the incorporation of “correct” dUTP opposite template A was lower (about 8-fold) than of dTTP with endogenous p53-harboring fractions (Figure 2A, lanes 7–12 and 2B).

Uracil may reside in DNA through the incorporation of dUTP instead of dCTP during replication, resulting in G_t_:U mispairs [25]. The insertion of correct dCTP or wrong dUTP opposite the template G was evaluated with mit(p53−/−) (Figure 2C, Seq II). The 17 mer product (about 4.5%) (Figure 2D) was generated after the production of correct C:G pair with no further extension with the time (Figure 2C, lanes 1–3). Noticeable, incorporation of dU and production of G_t_:U mispair was achieved at much higher nucleotide concentration of dUTP (1 mM) than the correct insertion of dCTP (50 μM) (Figure 2C, lanes 4–6). The experiments described in Figure 2C depicted that under the same experimental conditions a reduction occurs in the incorporation of the incorrect dU opposite the template G with mit(p53+/+) (lanes 10–12) relative to that detected with the incorporation of correct dCTP (lanes 7–8). A quantitative analysis reflects that the generation of G_t_:U mispair is about 8 times lower with mit(p53+/+) than with mit(p53−/−) (Figure 2D). Biochemical studies comparing the misincorporation of dU into DNA with p53-deficient mitochondrial lysates with those from isogenic p53-positive lysates, Showed that p53 may affect the pol γ selectivity for the incorporation of dU and production of both "correct" A_t_:U and “incorrect” G_t_:U pairs.

**Figure 3:**
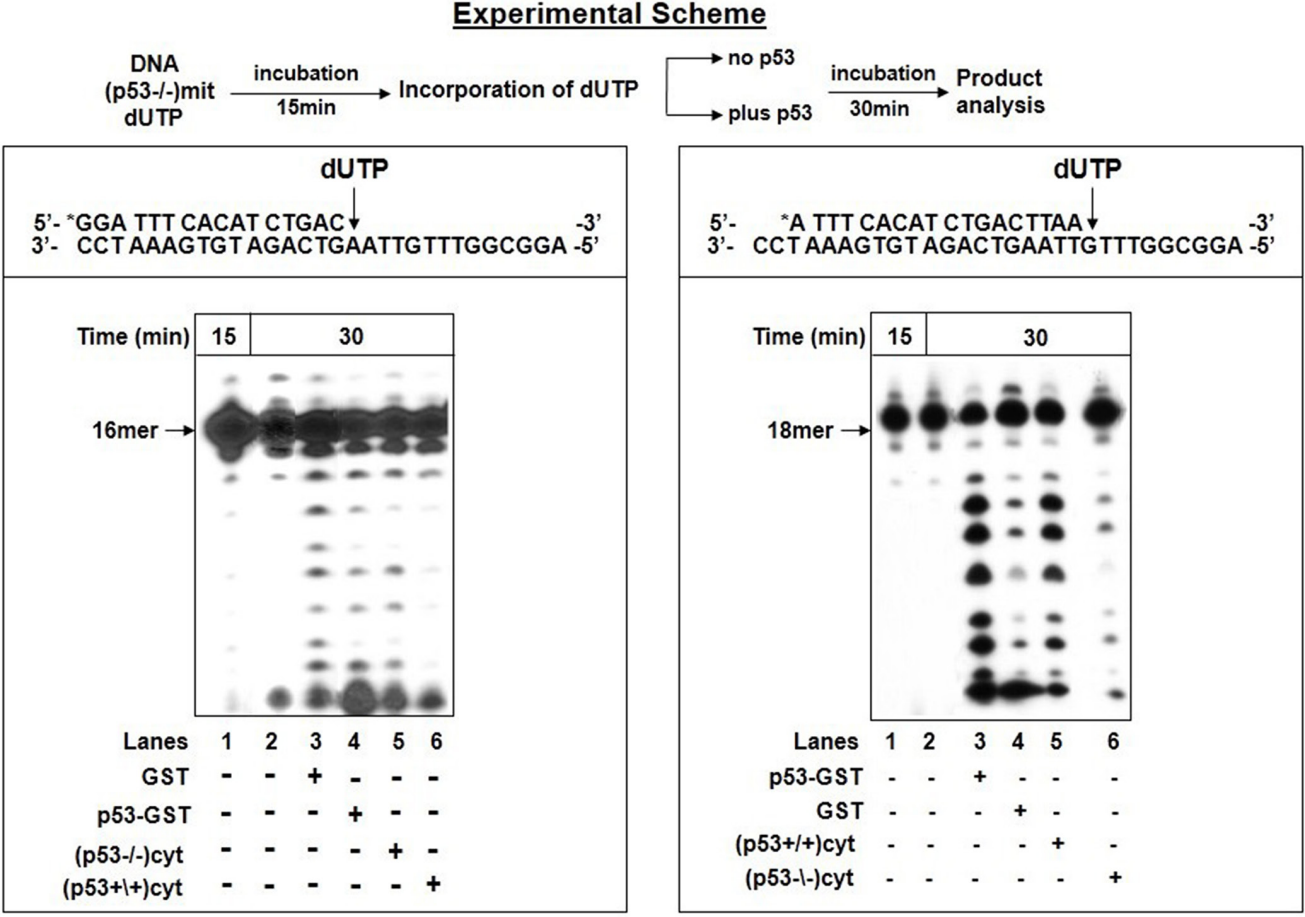
Excision of incorporated dUTP in the presence of p53 The excision of incorporated dUTP was tested according to the experimental scheme (top). After initial incubation of 5′-end labeled dsDNA substrate with dUTP and mit(p53−\−) for 15 min, aliquot was taken for analysis of incorporation of dUTP by PAGE (lane 1). The incubation was further extended in the absence or presence of p53. Left-Following the incorporation of dUTP opposite template A, the reaction mixture was further incubated for 30 min in the absence (lane 2) or presence of GST protein (lane 3) or p53-GST fusion protein (lane 4) or cytoplasmic fractions of (p53−\−) cells- cyt(p53−\−) (lane 5), or (p53+\+) cells- cyt(p53+/+) (lane 6). Aliquots were taken after additional 30 min incubation. Right-Following the incorporation of dUTP opposite template G, the reaction mixture was further incubated for 30 min in the absence (lane 2) or presence of p53-GST fusion protein (lane 3) or GST protein (lane 4) or cyt(p53+/+) (lane 5), or cyt(p53−\−) (lane 6). Aliquots were taken after additional 30 min incubation. The position of the 18 mer primer is indicated by an arrow.

The next challenge was to study the impact of the endogenous p53 on the excision of incorporated dU using a sequential reaction experiment, employed by us previously [22]. This strategy let to follow after the excision of the A_t_:U pair produced by pol γ in mit(p53−/−) in the absence or presence of either recombinant or endogenous p53 provided by cytoplasmic extracts of HCT116 cells (Figure 3). We took advantage of our recent observations that endogenous p53 in cytoplasmic extracts displays a high level of 3′→5′ exonuclease activity with dsDNA substrates [33]. After 15 min incubation, pol γ in mit(p53−/−) exerts dU insertion, thus generating 17- and 18 mer substrates for the excision step (Figure 3-left, lane 1). The accumulation of these products after additional 30 min incubation indicates that the A_t_:U pair was not efficiently corrected by an intrinsic excision activity of pol γ (lane 2). Conversely, following the misincorporation event the significant removal of the polymerization errors was witnessed in the presence of recombinant p53-GST fusion protein (lane 4), or endogenous p53-harboring cytoplasmic extracts [cyt(p53+/+)] (lane 6), as reflected in the decrease of the intensity of 17- and 18 mer products. In control experiments we verified no detectable editing of the wrong nucleotide after the supplementation of GST protein (lane 3) or cyt(p53−/−) (lane 5). We complemented this study by further characterizing the excision of incorrect G_t_:U pair in the presence of either recombinant (Figure 3-right, lane 3) or endogenous p53 (lane 5). These results further support the notion that the presence of p53 provides exonuclease activity enabling error-repair. It is highly likely that the error removal takes place by a direct excision mode implemented by external p53 exonuclease in mitochondria for processing of A_t_:U or G_t_:U pair.

### Proofreading in mitochondria

We performed two experiments to illustrate the proofreading potential of p53 as a trans-acting protein during error-correction process via dissociating-intermolecular mechanism (i.e. following the release of the DNA from the enzyme) in the mitochondria [34].

**Figure 4:**
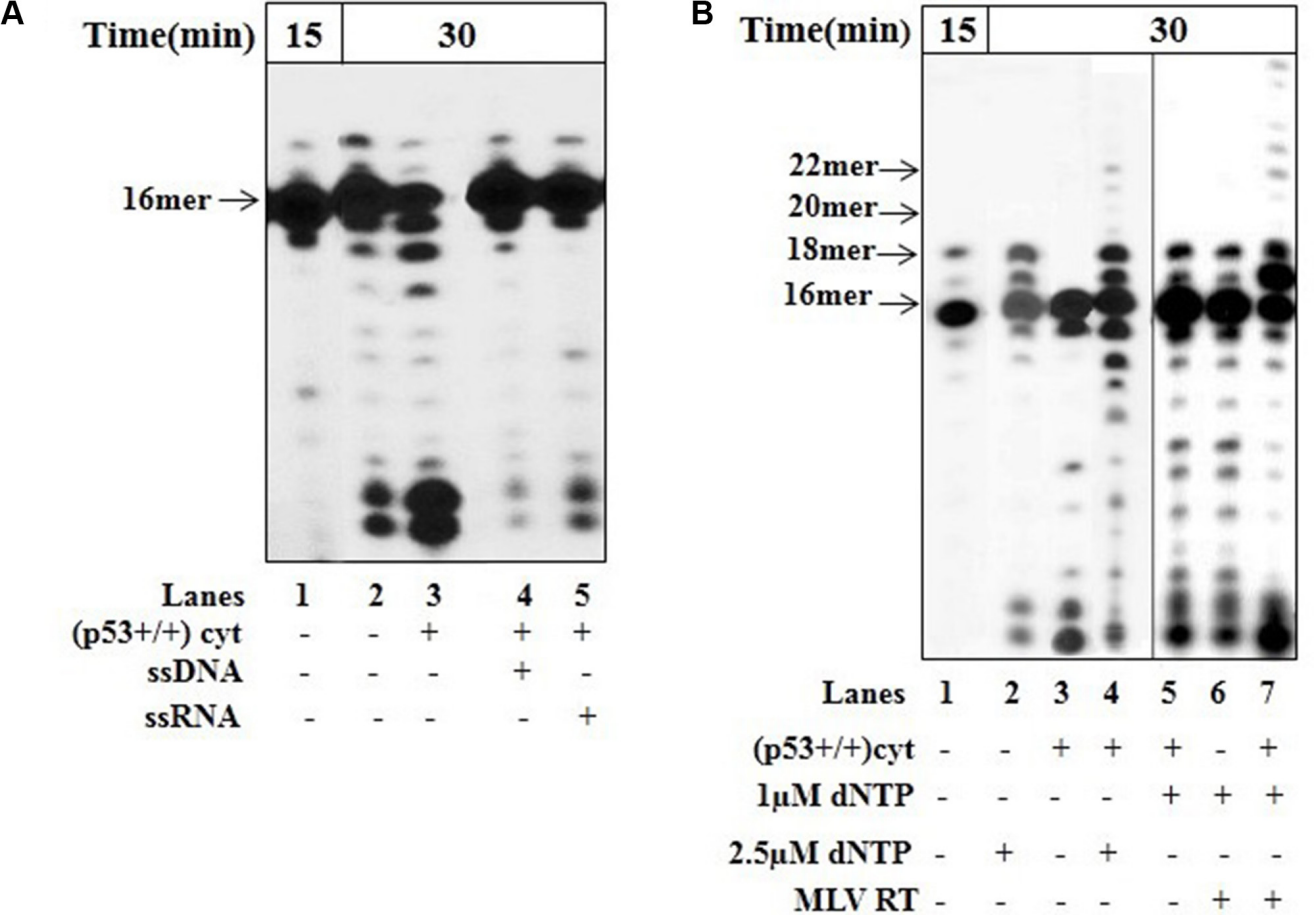
Error correction in the presence of p53 The incorporation of dUTP opposite the template A was examined with mit(p53−\−) in the presence of cyt(p53+\+) and unlabeled competitor DNA or RNA by using sequential experimental scheme, as described in Figure 3. (**A**) After initial incubation of dsDNA (Sequence of DNA-see Figure 3 left) for 15 min with mit(p53−\−) and incorporation of dUTP (lane 1), the reaction mixture was further incubated in the absence (lane 2) or presence of cyt(p53+\+) (lanes 3–5). Lane 3-incubation with cyt(p53+\+). Lane 4-incubation with cyt(p53+\+) and ssDNA. Sequence of the ssDNA trap-5′-ATTTCACATCTGACTA-3′. Lane 5-incubation with cyt(p53+\+) and ssRNA. Sequence of the ssRNA trap-5′-AUUUAUUUAUUAUUUUAUUAUU UAA-3′. Aliquots were taken after additional 30 min incubation. The position of the 16 mer primer is indicated by an arrow. (**B**) Functional interaction between DNA polymerase and endogenous p53 in mitochondrial fraction. After initial incubation of dsDNA (Sequence of DNA-see Figure 3-left) for 15 min with mit(p53−\−) and incorporation of dUTP (lane 1), the reaction mixture was further incubated in the presence of either 2.5 μM dNTP (lane 2), or cyt(p53+\+) (lane 3), or cyt(p53+\+) plus 2.5 μM dNTP (lane 4), or cyt(p53+\+) plus 1 μM dNTP (lane 5),or 1 μM dNTP plus MLV RT, or cyt(p53+\+) plus 1 μM dNTP plus MLV RT (lane 7). Aliquots were taken after additional 30 min incubation. The positions of the 16–22 mer primers are indicated by arrows.

To define if the excision of the newly produced A_t_:U pair includes the dissociation of dU-DNA from the DNA polymerase, we used a sequential reaction experiment. The reaction mixture following the initial incorporation of dU into DNA with mit(p53−/−) (Figure 4A, lane 1) was further incubated without (lane 2) or with cyt(p53+/+) in the absence (lane 3) or presence of unlabeled ssDNA (lane 4) or ssRNA (lane 5) trap. While incubation with cyt(p53+/+) alone results in the decrease in the level of 17- and 18 mer products (lane 3), the negative effect was decreased in the presence of ssDNA (lane 4) or ssRNA trap (lane 5). Obviously, the insertion of dU leads to the dissociation of the DNA-pol γ complex, thus assisting the dsDNA containing 3′-terminal A_t_:U pair to be reachable for editing by trans-acting exonuclease.

Proofreading is a multi-step process requiring the efficient excision of the wrong terminal nucleotide, the transfer of the primer to the polymerase for correct replacement of the incorrect nucleotide, and normal polymerization–primer elongation by DNA polymerase [5, 33]. The mode of action of p53 in error correction may be based on its direct interaction with DNA and biochemical (*e.g.* exonuclease) function. Since the p53 protein is a sequence-independent DNA-binding protein [35], it is highly likely that the physical binding of p53 to DNA may be a relevant event in the biological utility of the protein in DNA repair. Our recent studies, by analyzing the combined products of a DNA binding and polymerization reactions using sequential reaction experiment, revealed that within the context of error-correction events p53, by recognition and excision of 3′-mismatched nucleotides may be involved in DNA repair, thereby increasing the accuracy of DNA synthesis by DNA polymerases (*e.g.* HIV-1 RT) [35]. Here, we studied the functional interaction between p53 and pol γ, which is also a DNA binding protein. In sequential reaction experiment after the incorporation of dU by pol γ in mit(p53−/−) (Figure 4B, lane 1), no further elongation products were detected after the addition of dNTP at equimolecular concentrations (2.5 μM) (lane 2), indicating the inability of pol γ to further extend A_t_:U pair without correction of the error. However, the extension of mispaired substrates occurred by mit(p53−/−)/dNTP (2.5 μM) complex in the presence of cyt(p53+/+) resulting in the production of 19–22 mer products (lane 4). Particularly, the efficient excision of the incorporated A_t_:U mispair happens in the presence of cyt(p53+/+) alone (lane 3). We performed parallel experiments supplemented with other DNA polymerase, *e.g.* exonuclease-deficient murine leukemia virus (MLV) reverse transcriptase-RT. Indeed, the substrate was elongated by the MLV RT in the presence of 1.0 μM dNTP following the addition of cyt(p53+/+) giving rise to 19–27 mer products (lane 7). The fact that in the presence of 1.0 μM dNTP there was no detectable extension of the substrate by MLV RT alone (lane 6) or by pol γ even in the presence of cyt(p53+/+) (lane 5) further substantiate a functional cooperation of p53 exonuclease and polymerase in a coordinated manner during DNA synthesis [31]. The DNA elongation by either pol γ in mit(p53−/−) (lane 4) or by MLV RT (lane 7) occurs after initial correction of the terminal A_t_:U pair from un-extended substrate by p53, dissociation of the DNA from the protein, transfer to the polymerase (*e.g.* pol γ or MLV RT), and subsequent extension from the correctly paired 3′-terminus. Hence, we may conclude that p53 trans-acting protein in the mitochondria may contribute an exonuclease/proofreading function for removal of 3′-terminal dU by dissociating-intermolecular pathway during ongoing DNA synthesis.

### *In vivo* correlation between mitochondrial p53 and elevated exonuclease/proofreading activity

Several approaches were undertaken to evaluate the importance of endogenous p53 during the incorporation of dU.

**Figure 5:**
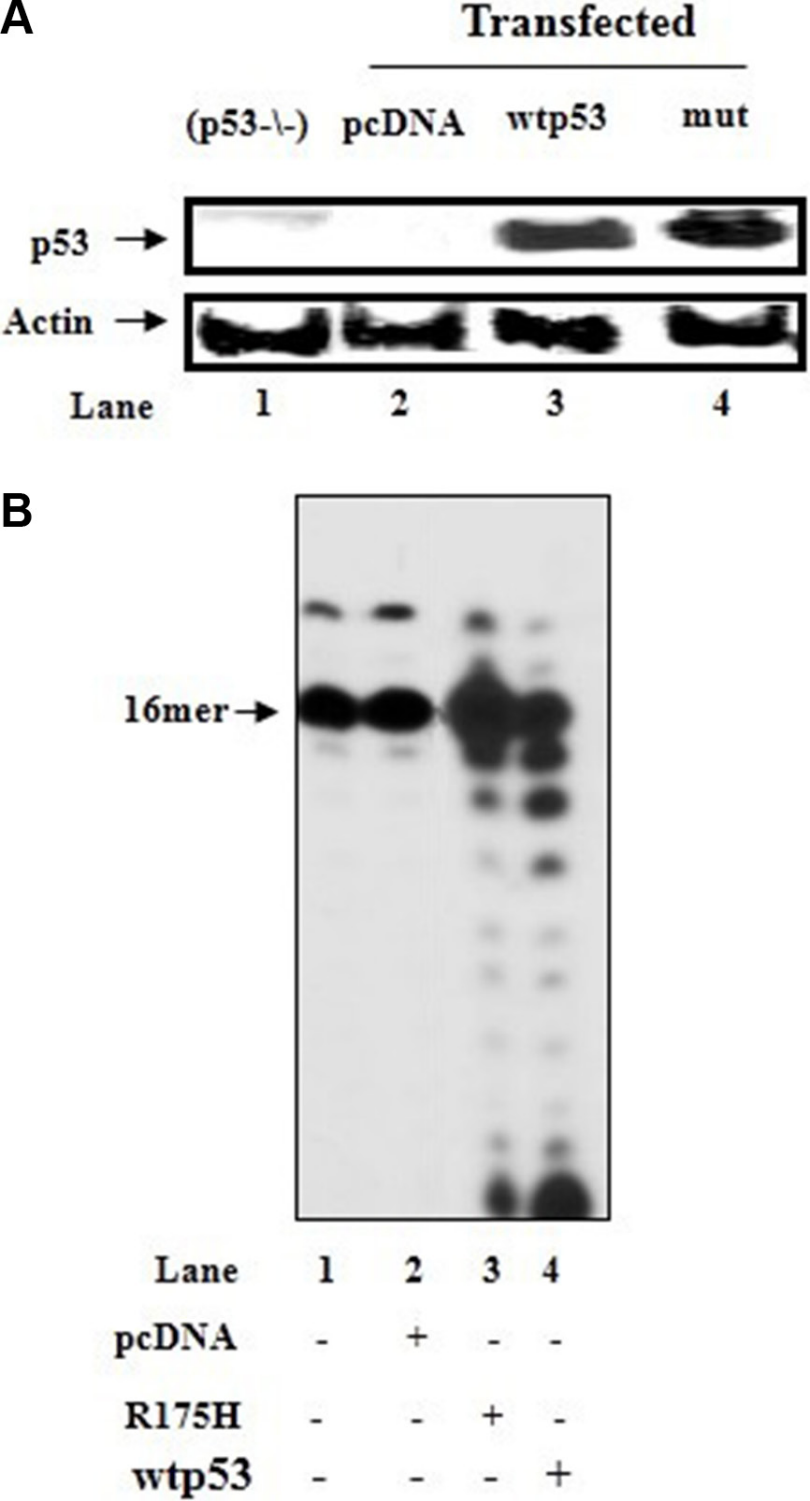
Incorporation of dUTP into DNA with mitochondrial fraction of HCT116(p53−/−) cells in the presence of cytoplasmic fractions of p53-transfected cells (**A**) Cytoplasmic lysates (10 μg) prepared from HCT116(p53−/−) cells (lane 1), or HCT116(p53−/−) cells transfected with the control plasmid (pcDNA) (lane 2) or with the wtp53 (lane 3) or mutant p53-R175H (mut) (lane 4) expression vector, were analyzed for p53 expression by immunoblotting. β-actin was blotted as a protein loading control. (**B**) After initial incubation of dsDNA substrate (Sequence of DNA-see Figure 3 left) for 15 min with HCT116(p53−/−)mit and incorporation of dUTP into DNA (lane 1), the reaction mixture was further incubated in the presence of cytoplasmic fractions (1 μg) of HCT116(p53−/−) cells transfected with control vector (lane 2) or wtp53 (lane 3) or mutant p53-R175H expression vector (lane 4). Aliquots were taken after 30 min incubation. The position of the 16 mer primer is indicated by an arrow.
- We assessed the impact of activated p53. Irradiation (IR) induces the mitochondrial translocation of p53 [37]. We focused on IR as HCT116 cells are resistant to apoptosis. We compared the expression levels of p53 protein and error-correction activities between the mitochondrial fractions of irradiated or untreated HCT116(p53+/+) cells. Mitochondrion-localized enhancement in the amount of endogenous p53 following the IR-stress signal, monitored by WB analysis (Figure 6A), coincides with an increase in constitutive excision capacity with 3′-terminal A:A containing dsDNA (Figure 6B, lanes 1–2) and low misincorporation of dU (Figure 6C). The fact that there was no significant difference in the correct polymerization activity between IR-exposed and control mit(p53+/+) (Figure 6B, lanes 3–4) points that pol γ itself was not responsible for the observed augmented error-correction activities in IR-mit(p53+/+); rather the elevation in the proofreading activities occurs through enrichment of mitochondrial p53.

- Our early studies demonstrated the efficient removal of the incorporated wrong nucleotide or nucleoside analog from DNA with cyt(p53−/−) overexpressing wtp53, but not with control lysates transfected with the vector or exonuclease function-deficient mutant p53-R175H [22, 36]. Here, we employed these cytoplasmic extracts to further explore the contribution of endogenous p53 to the A_t_:U mispir correction (Figure 5A). After the production of A_t_:U pair by pol γ with mit(p53−/−), the excision activity toward A_t_:U pair was more pronounced with cytoplasmic lysates obtained from (p53−/−) cells overexpressing wtp53 (Figure 5B, lane 4), compared with those cells transfected with either vector-pcDNA (lane 2) or mutant p53-R175H (lane 3).

**Figure 6:**
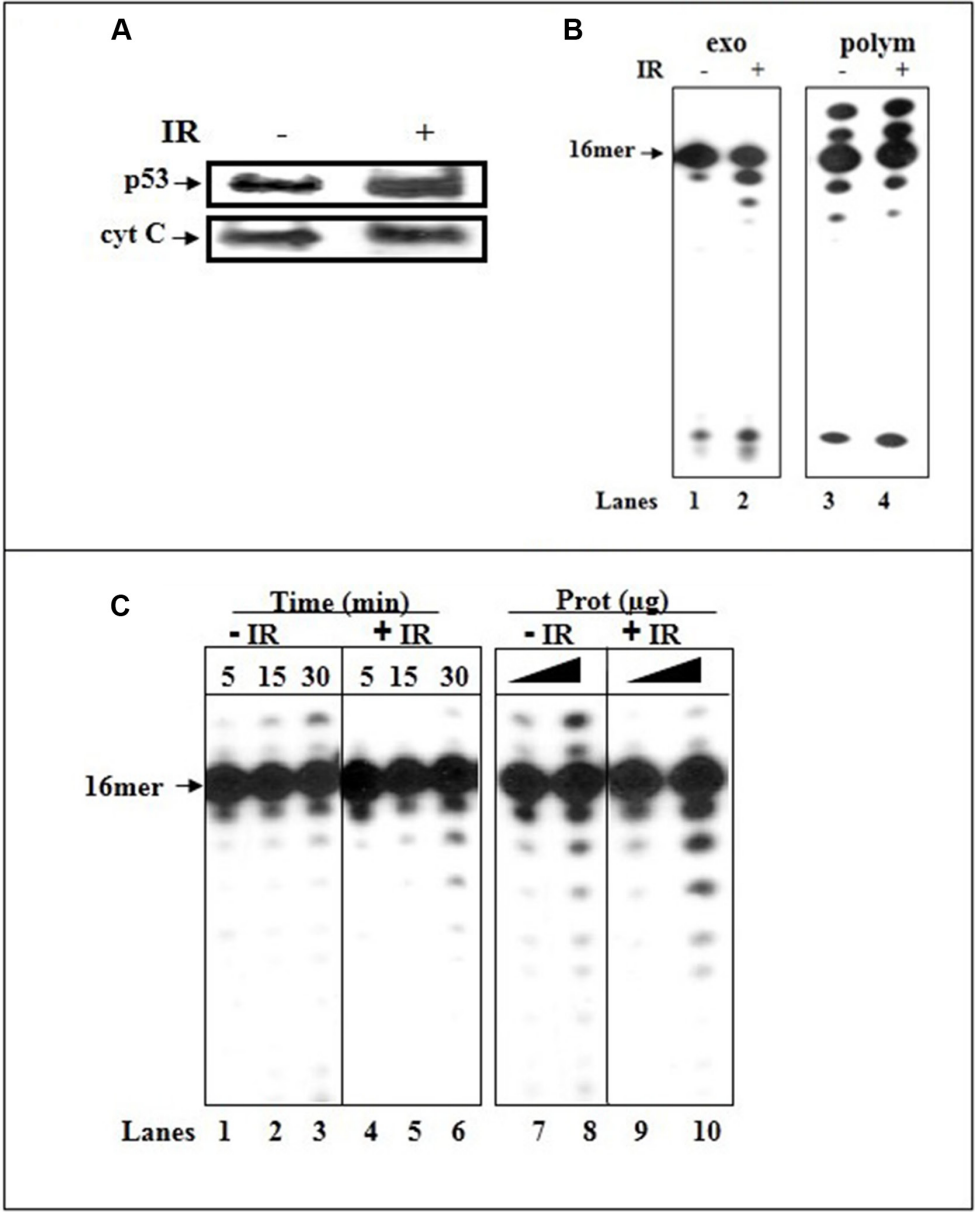
Irradiation induces translocation of p53 to mitochondria of HCT116(p53+/+) cells and increases the error-correction activities (**A**) Western blot analysis of mitochondrial fractions (20 μg) of untreated and IR-treated cells for the expression of p53. The mitochondrial protein cyt-c was used as a loading control. (**B**) The exonuclease activity (with dsDNA containing 3′-terminal A:A mispair) (lanes 1–2) or incorporation of correct dATP nucleotide (lanes 3–4) was examined with mitochondrial extracts of untreated (lanes 1 and 3, respectively) or IR-treated (24 hr post-IR) (p53+/+) cells (lanes 2 and 4, respectively). Sequence of template-primer-see Figure 1. (**C**) The incorporation of dUTP opposite the template A was examined with mitochondrial extracts of untreated (lanes 1–3 and 7–8) and IR-treated (24 hr post-IR) (lanes 4–6 and 9–10) HCT116(p53+/+) cells by increasing the incubation time (lanes 1–6), or the amount of mitochondrial protein extracts (lanes 7–10). Sequence of dsDNA -see Figure 3. Aliquots taken were analyzed by PAGE. The position of the 16 mer product is indicated by an arrow.
- Accumulation of p53 occurs upon activation by a various type of stress signals. Hence, we further explored the significance of activated p53 induced by drug treatment. Nutlin is a competitive inhibitor of the MDM2-p53 interaction that stabilizes and activates p53 in the absence of genotoxic stress to a degree comparable to genotoxic p53 activation [38]. Nutlin treatment causes cytoplasmic p53 accumulation and translocation to mitochondria with subsequent transcription-independent functions associated with p53-mediated cell death [39]. We have examined the mitochondrial lysates of (p53+/+) cells exposed for 6 hr to nutlin (10 μM) for p53 expression and error-correction activities. In agreement with the reported results we confirmed the nutlin-induced mitochondrial p53 translocation by WB analysis (Figure 7A). The assessment of these fractions for exonuclease activity revealed an increase in constitutive excision proficiency with dsDNA primer-templates containing 3′-terminal A:A mispair with nutlin-treated mit(p53+/+) relative to untreated lysates, in accordance with the enhancement in the amount of endogenous p53 (Figure 7B, lanes 4–5). In control experiments, we detected no increase in removal of the mispair in mitochondrial fractions of nutlin-treated (p53−/−) cells (Figure 7B, lanes 2–3). Furthermore, the decline in incorporation of dU into DNA was detected with nutlin-treated mit(p53+/+) compared to that of untreated mit(p53+/+), indicating much stronger negative effect (Figure 7C, lanes 1 and 2). Pifithrin μ (PFT μ), a cell-permeable specific inhibitor of p53 translocation to mitochondria substantially reduced nutlin-induced apoptosis [38]. Hence, we utilized this agent to further underline the significance of p53 in DNA repair program during the incorporation of dU into DNA. Indeed, mitochondrial fractions of (p53+/+) cells pretreated with PFT μ prior to nutlin exposure showed detectable decrease in the level of p53 in mitochondria (Figure 7A), in mispair excision ability (Figure 7B, lane 6) coincident with low efficiency of prevention of dU incorporation, compared to nutlin-treated lysates (Figure 7C). These results support the view that activated p53 in mitochondria may provide an intrinsic error-correction activities for DNA damage repair during incorporation of dU.

**Figure 7:**
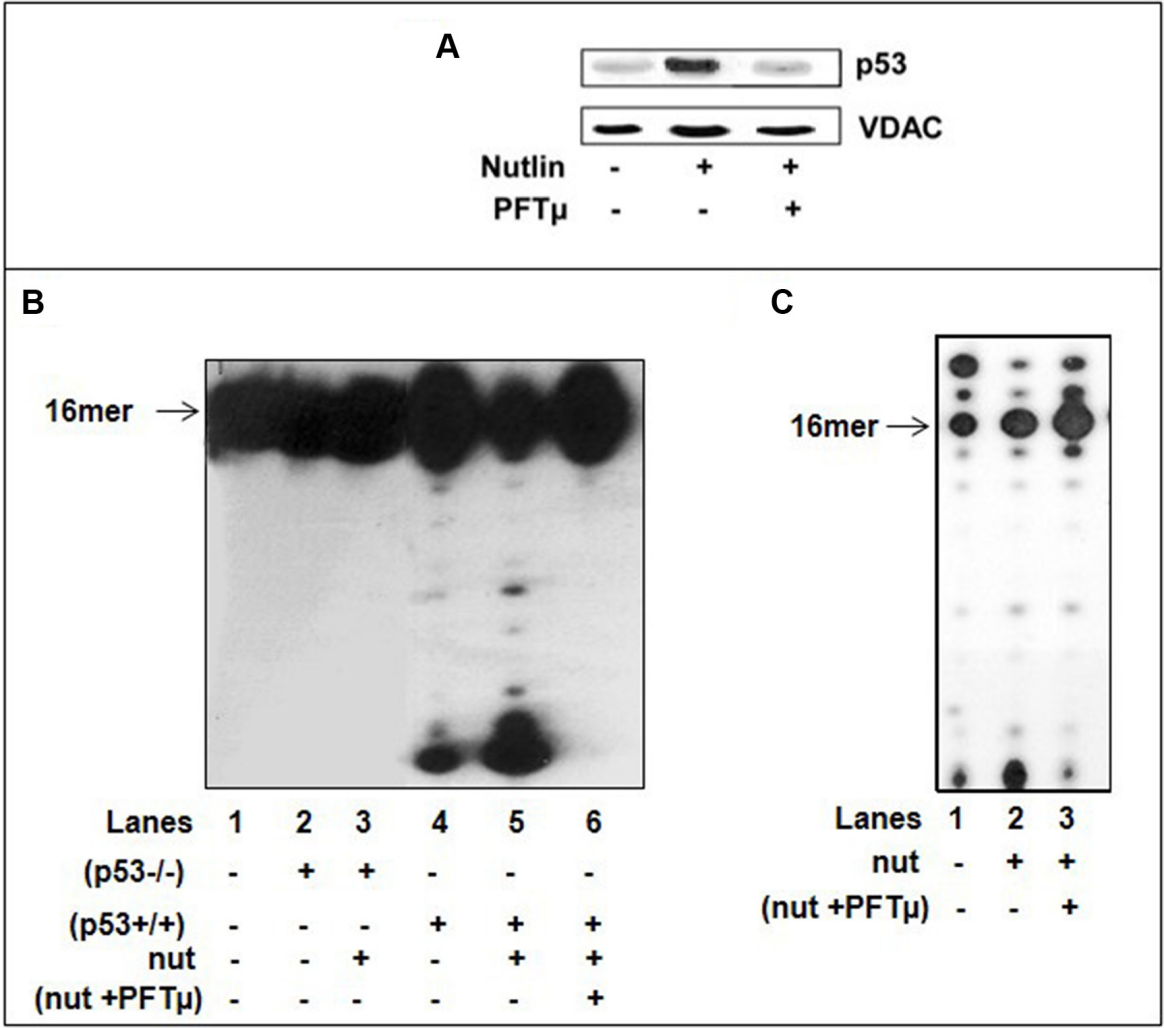
Nutlin treatment induces translocation of p53 to mitochondria of HCT116(p53+/+) cells and decreases the incorporation of dU into DNA (**A**) Western blot analysis of mitochondrial fractions (5 μg) of untreated, nutlin-treated 10 μM nutlin for 6 hrs or cells pre-treated with 25 μM PFTμ for 2 hrs before adding nutlin for the expression of p53. The mitochondrial protein VDAC was used as a loading control. (**B**) The exonuclease activity with dsDNA containing 3′-terminal A:A mispair (lane 1) was analyzed with mitochondrial fractions of HCT116(p53−/−) cells nutlin-untreated (lane 2), nutlin-treated cells (10 μM for 6 hr) (lane 3) or mitochondrial fractions of (p53+/+) cells nutlin un-treated cells (lane 4), or nutlin-treated cells (10 μM for 6 hr) (lane 5) or (p53+/+) cells pre-treated with 25 μM PFTμ for 2 hrs before adding nutlin (lane 6). Sequence of template-primer-see Figure 1B. (**C**) The incorporation of dUTP opposite the template A was examined with mitochondrial extracts of untreated (lane 1), nutlin-treated (p53+/+) cells (10 μM for 6 hr) (lane 2) or (p53+/+) cells pre-treated with 25 μM PFTμ for 2 hrs before adding nutlin (lane 3). Sequence of dsDNA -see Figure 1C. Aliquots taken were analyzed by PAGE. The position of the 16 mer product is indicated by an arrow.

## DISCUSSION

Mammalian cells have established a diverse defense network to safeguard genomic integrity [2]. The aim of the current study was to test the hypothesis that p53 could reduce the incidence of production mutagenic uracil damage in mtDNA. Our biochemical studies demonstrate that mitochondrial p53 possesses the potential to diminish the incorporation of non-canonical dUTP into DNA by pol γ. Evidence that enhanced exonuclease/proofreading function for dU damage repair detected in mitochondrial extracts is assigned to p53 are supported by numerous experimental evidences. 1) Endogenous p53 has an impact on the replication accuracy of pol γ during the incorporation of dU into DNA according to two criteria: a) The presence of p53 reduced the efficiency of dU incorporation into DNA, although p53 status did not affect the insertion of correct nucleotide. The significance of p53 is supported by the immunodepletion of p53 from mitochondrial extracts of repair-proficient p53-harboring cells diminishing this negative effect. b) The procession of “correct” A_t_:U and “mismatched” G_t_:U lesions enhances in the presence of recombinant or endogenous wtp53. 2) Within the context of error-correction events, p53 as a DNA binding protein, contributes an external proofreading function; upon excision of the dU, the p53 dissociates, thus letting the transfer of the substrate with the correct 3′-terminus to DNA polymerase and renewal of DNA synthesis. 3) Increased removal of incorporated dU was witnessed in mit(p53−/−) by complementation of exonuclease activity executed by cyt(p53−/−) overexpressing wtp53, but not exonuclease-deficient mutant p53-R175H. 4) Augmented abundance of p53 in IR-mit(p53+/+) or nutlin-treated mit(p53+/+) caused in a decreased dU incorporation. Altogether, the data corroborate the significance of p53 as an exonuclease for the excision of non-canonical dUTP, thus expanding the spectrum of DNA damage sites exploited for proofreading as a trans-acting protein. The fact that p53 may improve the accuracy of DNA synthesis by the decline of dU incorporation capacity plus excision from the nascent strand, infers that p53 potentially may have an additional editing activity in error-correction pathways thus determining replication accuracy and preservation of genome integrity.

p53 exhibits the functional heterogeneity in its basal (non-induced) state and under various p53 inducible circumstances [9, 10]. p53 unveils pro-apoptotic and pro-survival functions thereby triggering mitochondrial-directed apoptosis and mtDNA repair [13, 14]. The dichotomy in p53 action is dictated by its level of expression and activity, and the availability of its interacting partners. The data attained raise questions about the possible functional implication of p53-exonucleolytic proofreading activity in the mitochondria of normal and tumor cells.

- The abnormal accumulation of dUTP with subsequent incorporation into DNA depends on the local intracellular dUTP/dTTP ratio [40]. The amount of dU remaining in DNA may be low due to efficient dU removal and repair. In differentiated, non-proliferating cells, *e.g.* neurons, quiescent lymphocytes and macrophages the intracellular nucleotide pool might be imbalanced with much higher levels of dUTP [41]. Under physiological non-stressed conditions, the maintenance of uracil-free DNA is achieved through the combined actions of two enzymes dUTPase and UDG [27]. The fact that p53 provides a proofreading function for exonuclease-deficient as well as –proficient polymerase, suggests that p53 in normal cells, as a trans-acting protein, can both eliminate dU insertion into DNA and repair the mistake by excision of the incorporated dU from nascent DNA. Since dUTP is known to cause DNA damage and cell death when it is incorporated into DNA, p53 by protective activities can contribute pro-survival function.
- Various stresses, such as DNA damage, oncogene expression, can trigger the translocation of p53 to mitochondria in many primary and some transformed cell types [41, 42]. We speculate that if dUTPase activity could be impaired, under conditions that provoke dUTP accumulation and enhanced misincorporation into DNA, p53 exonuclease-proofreading activity might avoid mtDNA damage produced by the dUTP–DNA misincorporation pathway. Remarkably, the level of dUTP increases in response to certain stress signals [43]. Enhanced error-correction responses were observed following the IR stress-facilitated mitochondrial localization of p53, implying the increased DNA repair potential of p53 in stressed cells.
- Different stimuli will lead to the production of different ROS species and in turn these will elicit different responses [42]. The cellular mechanisms responsible for preventing cell death in response to stress signals and to the activation of apoptotic pathways represent complex and diverse mechanisms [44]. Recent studies have suggested that there is a cross-talk between the processes involved in regulating the levels of ROS and dUTP [44]. The accumulated mtDNA mutations are sourcing of augmented levels of reactive oxygen species ROS, which leads to enhanced mitochondrial and nuclear DNA injury. MtDNA damage enlarges the ROS creation formed by mtDNA mutations, probably through transcription mutagenesis, implying conversion of damaged DNA into mutated transcripts further translated into mutated mitochondrial proteins. Nuclear DNA damage facilitates translocation of p53 to the nucleus which decreases the availability of p53 pool in mitochondria. It is conceivable to hypothesize that under conditions of low levels of dUTP residual p53 may exert error-correction activities, while in the presence of elevated dUTP levels, the shift from repair to apoptosis may happen.
- Antimetabolites, widely used in the treatment of a broad range of neoplastic diseases, are highly toxic to normal cycling cells due to the misinsertion of dU into DNA, and can potentially induce the development of secondary tumors [28]. In the presence of 5-FU metabolites, the efficiency of dUTPase diminished [45]. The combined cytotoxicity from simultaneous dTTP-pool depletion, excessive amounts of dUTP and dU misincorporation can result in enhanced DNA damage and cell death [29]. p53 potentially can defend cells from negative consequences of dUTP pool expansion with consequent dU misincorporation into DNA.
- p53 is involved in the downstream signaling in response to 5FU [30]. The induction of p53 in response to 5-FU is a later event; 5-FU treatment did not promote accumulation of p53 until 24 h post-treatment [46]. Furthermore, the down-regulation of dUTPase during apoptosis was reported suggesting that dUTPase expression can be modulated by the p53 [46]. Tumoral p53 status and mutation may be important regulators of dUTPase expression, thereby affecting chemotherapeutic response [45, 47]. It is plausible that the p53-mediated repression of dUTPase which enhance DNA damage signifies the importance of this component in cell death thus contributing to the tumor suppressing functions directed by p53 [30]. The shift from repair to apoptosis can occur when DNA damage overwhelms repair capacity or following the interaction of p53 with other pro-apoptotic proteins. These routes need to be further explored through complementary experiments.

In sum, the current results suggest that p53 in mitochondria may facilitate mtDNA damage repair functions resulting from uracil–DNA misincorporation.

## MATERIALS AND METHODS

### Cell lines and culture media

The colorectal cancer cells isogenic for p53, HCT116(p53+/+) and HCT116(p53−/−), were grown in McCoy's medium supplemented with 10% FBS. HepG2 cells were grown in EMEM (ATCC, Manassas, VA, USA) with 10% FBS (ATCC) and penicillin/streptomycin (Invitrogen). Cells were maintained at 37°C in a humidified 95% air, 5% CO_2_ atmosphere.

### Preparation of mitochondrial fractions

Mitochondria were prepared as described [22].

### Western blotting

Equal amounts of total protein of mitochondrial, nuclear or cytoplasmic fractions were subjected to WB analysis as described [22]. After transferring proteins onto nitrocellulose, the blots were probed with the indicated antibodies, and protein bands were developed by using chemiluminescence. The following antibodies were used: monoclonal antibodies for p53 (Do-1) (Oncogene), for cytochrome-c (Oncogene), for VDAC (Santa Cruz Biotechnology) and polyclonal antibody for c-jun (Santa Cruz Biotechnology).

### Exonuclease/polymerase coupled assays

The DNA primer extension assay was used allowing simultaneous detection of both, degradation and extension. The sequence of the template-primers used for the experiments are depicted in figures. The primers end labeled at the 5′-end with T4 polynucleotide kinase (Fermentas) and [γ-^32^P] ATP were annealed to the template DNA as described [31]. The incubation mixture (10 μl) contained 50 mM Tris HCl (pH 7.5), 5 mM MgCl_2_, 1 mM DTT, 0.1 mg/ml BSA, 5′-end labeled substrates, nucleotides and mitochondrial protein extracts. The reaction products (polymerization or excision) were analyzed by electrophoresis through 16% polyacrylamide gel electrophoresis (PAGE) and detected by autoradiography, as described [22]. The extended and excised products were quantified by densitometric scanning of gel autoradiographs and the percentage of the total amounts of primers extended or excised was calculated.
